# DYRK1B as therapeutic target in Hedgehog/GLI-dependent cancer cells with Smoothened inhibitor resistance

**DOI:** 10.18632/oncotarget.6910

**Published:** 2016-01-13

**Authors:** Wolfgang Gruber, Martin Hutzinger, Dominik Patrick Elmer, Thomas Parigger, Christina Sternberg, Lukasz Cegielkowski, Mirko Zaja, Johann Leban, Susanne Michel, Svetlana Hamm, Daniel Vitt, Fritz Aberger

**Affiliations:** ^1^ Cancer Cluster Salzburg, Department of Molecular Biology, University of Salzburg, Salzburg, Austria; ^2^ 4SC Discovery GmbH, Planegg-Martinsried, Germany; ^3^ Department of Pediatrics, Medical University of Vienna, Vienna, Austria; ^4^ 4SC AG, Planegg-Martinsried, Germany

**Keywords:** Hedgehog/GLI signaling, GLI transcription factors, DYRK1B, Smoothened drug resistance, basal cell carcinoma

## Abstract

A wide range of human malignancies displays aberrant activation of Hedgehog (HH)/GLI signaling, including cancers of the skin, brain, gastrointestinal tract and hematopoietic system. Targeting oncogenic HH/GLI signaling with small molecule inhibitors of the essential pathway effector Smoothened (SMO) has shown remarkable therapeutic effects in patients with advanced and metastatic basal cell carcinoma. However, acquired and *de novo* resistance to SMO inhibitors poses severe limitations to the use of SMO antagonists and urgently calls for the identification of novel targets and compounds.

Here we report on the identification of the Dual-Specificity-Tyrosine-Phosphorylation-Regulated Kinase 1B (DYRK1B) as critical positive regulator of HH/GLI signaling downstream of SMO. Genetic and chemical inhibition of DYRK1B in human and mouse cancer cells resulted in marked repression of HH signaling and GLI1 expression, respectively. Importantly, DYRK1B inhibition profoundly impaired GLI1 expression in both SMO-inhibitor sensitive and resistant settings. We further introduce a novel small molecule DYRK1B inhibitor, DYRKi, with suitable pharmacologic properties to impair SMO-dependent and SMO-independent oncogenic GLI activity. The results support the use of DYRK1B antagonists for the treatment of HH/GLI-associated cancers where SMO inhibitors fail to demonstrate therapeutic efficacy.

## INTRODUCTION

The Hedgehog (HH)/GLI pathway plays a central role in the control of vertebrate development and tissue homeostasis of adult mammalian organisms, while its uncontrolled activation or inefficient termination has been implicated in a number of human malignancies including cancers of the skin, brain, hematopoietic system, lung, ovary and of the gastrointestinal tract [1]. Targeting oncogenic HH/GLI signaling in cancer and cancer stem cells has therefore emerged as promising therapeutic strategy for many malignant diseases with high medical need [2, 3].

Precise control of canonical HH/GLI signaling is an intricate process involving numerous regulatory processes. Briefly, in the absence of HH ligand, the twelve-transmembrane protein Patched (PTCH) represses HH signaling by preventing the G-protein coupled receptor-like protein Smoothened (SMO) to translocate to the primary cilium, an antenna-like compartment central to the activation of canonical HH/GLI signaling. Binding of HH to its receptor PTCH revokes the repressive effect of PTCH thereby allowing SMO to enter the primary cilium and initiate signaling. In the cilium, SMO activates the GLI zinc finger transcription factors GLI2/3 by preventing the formation of GLI repressor forms and by releasing GLIs from their negative regulator Suppressor of Fused (SUFU). Translocation of GLI2/3 activator forms to the nucleus induces HH/GLI target gene expression, including the potent transcriptional activator and oncogene GLI1 (for detailed reviews see [4-7]).

The etiologic role of HH signaling in cancer has triggered numerous efforts to develop HH pathway antagonists targeting the essential HH effector SMO [8]. In 2012, the FDA approved vismodegib, a small molecule inhibitor of SMO, for the treatment of non-melanoma skin cancer. Vismodegib provides a remarkable therapeutic benefit to patients with advanced or metastatic basal cell carcinoma (BCC) [3], a malignancy caused by loss of PTCH or mutational activation of SMO [9]. Despite several successful trials with BCC patients and case studies with medulloblastoma (MB) patients, the therapeutic efficacy of SMO targeting is challenged by acquired and *de novo* drug resistance [10-12]. Furthermore, clinical trials with SMO inhibitors so far have failed to prove a clear therapeutic benefit for patients with non-BCC malignancies including colorectal, ovarian and pancreatic cancer [13, 14].

*De novo* resistance to SMO targeting can – at least in part – be explained by the uncoupling of GLI activation from canonical SMO-dependent HH signaling. Various molecular cues and genetic alterations responsible for SMO-independent GLI activation in cancer cells have been identified. Oncogenic receptor tyrosine kinases, RAS/MAP kinase, PI3K/AKT/S6K, DYRK1A, PKC and histone deacetylases can enhance the transcriptional activity of GLI in human cancer cells [15-21]. Likewise, genetic loss of SUFU results in constitutive GLI activation independent of SMO signaling [22]. In pancreatic cancer, TGFβ/SMAD signaling is able to induce expression of GLI activator forms [23] and in Ewing Sarcoma the EWS-FLI1 oncogene directly stimulates GLI1 expression [24].

GLI proteins, particularly GLI1, act as potent oncogenic drivers by promoting a variety of malignant traits including proliferation, survival, invasion and metastasis (reviewed in [7]). GLI1 also represents a critical determinant of tumor-initiating cancer stem cells in several entities such as glioblastoma, colorectal cancer and pancreatic cancer [16, 25-27]. These oncogenic properties together with the capacity of GLI1 to integrate and relay common SMO-independent cancer-promoting cues such as receptor-tyrosine kinase pathways, PI3K and MAP kinase signaling render GLI1 an attractive molecular target for cancer therapy. However, unlike kinase inhibition, direct targeting of transcription factors is generally considered challenging. Some recent studies demonstrated successful inhibition, though with yet unclear clinical relevance and specificity [28-32].

**Figure 1:**
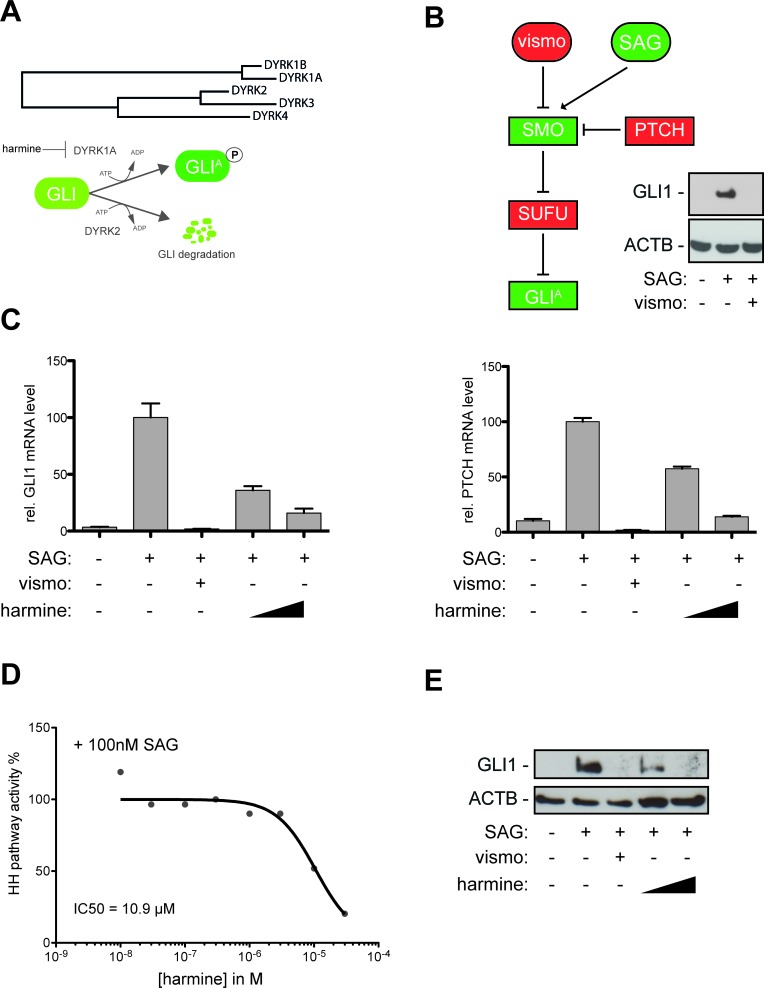
The DYRK1 inhibitor harmine blocks canonical HH/GLI signaling **A**. Evolutionary distance of DYRK family members and mode of action of distinct DYRK members on GLI activation (DYRK1A) and GLI degradation (DYRK2). **B**. DAOY human medulloblastoma cells harbor a responsive canonical HH/GLI signaling system. Treatment with the SMO agonist SAG (100nM) results in activation of GLI1 expression that is quantitatively abolished in the presence of the clinically approved SMO inhibitor vismodegib (vismo) (0.5 μM). Treatment with recombinant sonic HH protein yielded comparable results (data not shown). **C**. qPCR analysis showing repression of GLI1 mRNA (left) and PTCH mRNA expression (right panel) in SAG-stimulated DAOY cells in response to vismodegib (0.5 μM) or harmine treatment (10 μM and 20 μM). **D**. Analysis of concentration-dependent inhibition of HH pathway activity and IC_50_ calculation of 10.9 μM for the natural DYRK inhibitor harmine. **E**. Efficient inhibition of GLI1 protein expression in SAG-stimulated DAOY cells either treated with vismodegib (0.5 μM) or harmine (10 μM and 20 μM).

We therefore turned our focus to kinases as well established therapeutic targets to identify druggable effectors involved in promoting both canonical and SMO-independent GLI activation in cancer. Candidate kinases include members of the Dual-Specificity Tyrosine-Phosphorylation-Regulated Kinase (DYRK) family, which have been shown to positively and negatively modify HH signaling and to have oncogenic functions in solid cancers known to be associated with HH/GLI signaling including pancreatic cancer [33]. The DYRK family comprises two subfamilies with a total of five members [34]. Of note, the class I DYRK family member DYRK1A is able to enhance GLI1 activity, while the closely related yet functionally distinct class I member DYRK1B has been shown to increase HH ligand expression and prevent autocrine HH pathway activation [15, 35]. By contrast, the class II family member DYRK2 negatively affects HH/GLI signaling by triggering the destabilization and degradation of GLI2/3 transcription factors (Figure 1A) [36]. Whether DYRK family members can serve as therapeutic targets in HH/GLI-associated cancer entities has not yet been addressed.

In this study we analyzed the role of class I DYRK members and identified DYRK1B as critical player in both SMO-inhibitor sensitive and resistant settings. Furthermore, we introduce a novel small molecule DYRK1B inhibitor with potent *in vitro* and *in vivo* activity targeting GLI dependent cancer cells. We propose that small molecule inhibition of DYRK1B represents a novel and promising approach to target HH/GLI-associated cancers including malignancies with acquired or *de novo* resistance to SMO inhibitors.

## RESULTS

### Chemical inhibition of class I DYRK members impairs HH/GLI pathway activation

Members of the DYRK family can modulate GLI activity in opposite directions. While DYRK2 promotes GLI degradation [36], overexpression of the class I family member DYRK1A is able to promote the transcriptional activity of GLI1 (Figure 1A) [15]. Whether small-molecule modulation of DYRK is able to inhibit HH/GLI signaling in cancer cells has not yet been addressed.

To study whether inhibition of DYRK1 kinases affects oncogenic HH/GLI signaling, we first measured the effect of the known class I DYRK inhibitor harmine [37] on HH/GLI pathway activity. As assay system we employed HH-responsive, SMO-inhibitor sensitive human medulloblastoma cells (DAOY) (Figure 1B) [38] and measured changes in the expression of the known HH target genes GLI1 and PTCH as quantitative read-out for pathway activity.

Smoothened Agonist (SAG) treatment of DAOY cells induced mRNA expression of the HH targets GLI1 and PTCH, which was effectively repressed by the SMO inhibitor vismodegib (vismo) and notably, also by the DYRK1 inhibitor harmine in a concentration dependent manner with an IC_50(harmine)_ of 10.9 μM (Figure 1C, 1D). Similar to vismodegib, harmine prevented the accumulation of GLI1 protein in response to SAG treatment (Figure 1E), together suggesting that class I DYRK family members may serve as targets for HH/GLI signal inhibition by non-SMO antagonists.

### Identification of DYRK1B as critical effector in canonical HH/GLI signaling

**Figure 2:**
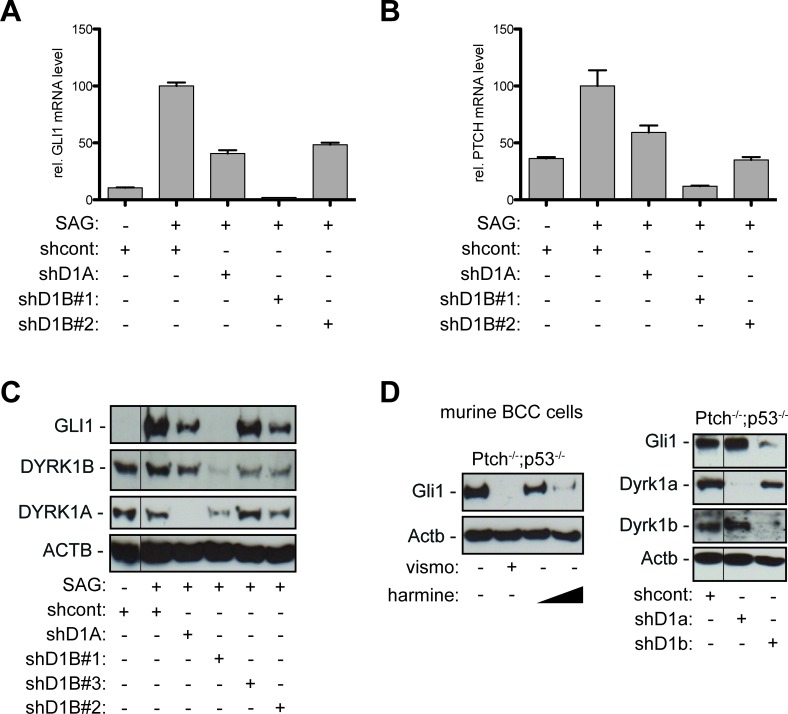
Genetic perturbation of DYRK1B interferes with canonical HH/GLI pathway activation **A**.-**B**. qPCR analysis of GLI1 (A) or PTCH mRNA expression (B) in SAG-treated DAOY cells stably transduced with scrambled control shRNA (shcont), shRNA against DYRK1A (shD1A), or two shRNAs against DYRK1B (shD1B#1, shD1B#2). **C**. Western blot analysis of GLI1 protein expression in SAG-stimulated DAOY cells expressing the respective lentiviral shRNA constructs and a third shRNA against DYRK1B (shD1B#3). **D**. Western blot analysis of Ptch-deficient murine BCC cell lines showing abrogation of Gli1 expression by harmine treatment (left panel) (10 μM and 20 μM) and by shRNA against Dyrk1b (shD1b) but not by shRNA against Dyrk1a (shD1a) (right panel). Fine black lines indicate cropping of intermediate lanes from the same Western blots. ACTB/Actb: human/mouse beta actin loading control.

The beta-carboline alkaloid harmine is a potent inhibitor of both class I family members DYRK1A and DYRK1B and of monoamine oxidase-A [37, 39]. To validate the involvement of DYRK1 kinases in HH pathway regulation and to address whether DYRK1A, DYRK1B or both kinases account for the repressive effect of harmine on HH/GLI pathway activity, we performed genetic RNA-interference mediated perturbation experiments in cells with activated canonical HH/GLI signaling. In SAG-stimulated human medulloblastoma cells, stable, lentiviral shRNA inhibition of DYRK1A moderately reduced GLI1 and PTCH mRNA expression by 55 and 40 percent, respectively (Figure 2A, 2B). By contrast, knockdown of DYRK1B efficiently abolished HH target gene expression below levels of unstimulated cells. We confirmed the contribution of DYRK1B to HH target gene activation with a second, less functional shRNA against DYRK1B (shD1B#2) resulting in reduction of GLI1 and PTCH mRNA expression by more than 50 and 60 percent, respectively, compared to SAG-stimulated cells transduced with scrambled control shRNA (Figure 2A, 2B). Analysis of GLI1 protein expression confirmed mRNA expression data. Inhibition of DYRK1A moderately reduced GLI1 protein levels in SAG-treated DAOY cells, while depletion of DYRK1B efficiently eliminated GLI1 protein expression (Figure 2C). Reduction of DYRK1B expression using shD1B#2 with moderate or shD1B#3 with little functionality repressed GLI1 protein expression proportional to the shRNA functionality (Figure 2C). RNAi-mediated inhibition of DYRK1B did not impair the formation of the primary cilium, an antenna-like compartment essential for HH/GLI signal transduction [4], suggesting that HH/GLI inhibition by DYRK1B targeting is not due to impaired ciliogenesis (Figure S1). The requirement of Dyrk1b for Hh/Gli signaling was also obvious in murine Ptch-deficient BCC cells [40]. Harmine treatment of murine BCC cells reduced Gli1 expression in a concentration-dependent manner (Figure 2D and Figure S2), and like in human medulloblastoma cells, RNAi mediated perturbation of Dyrk1b efficiently inhibited Gli1 protein expression, while depletion of Dyrk1a did not. Taken together, these data identify DYRK1B as druggable target for the inhibition of oncogenic HH/GLI signaling.

### Targeting DYRK1B inhibits HH/GLI signaling in SMO-inhibitor resistant cells

**Figure 3:**
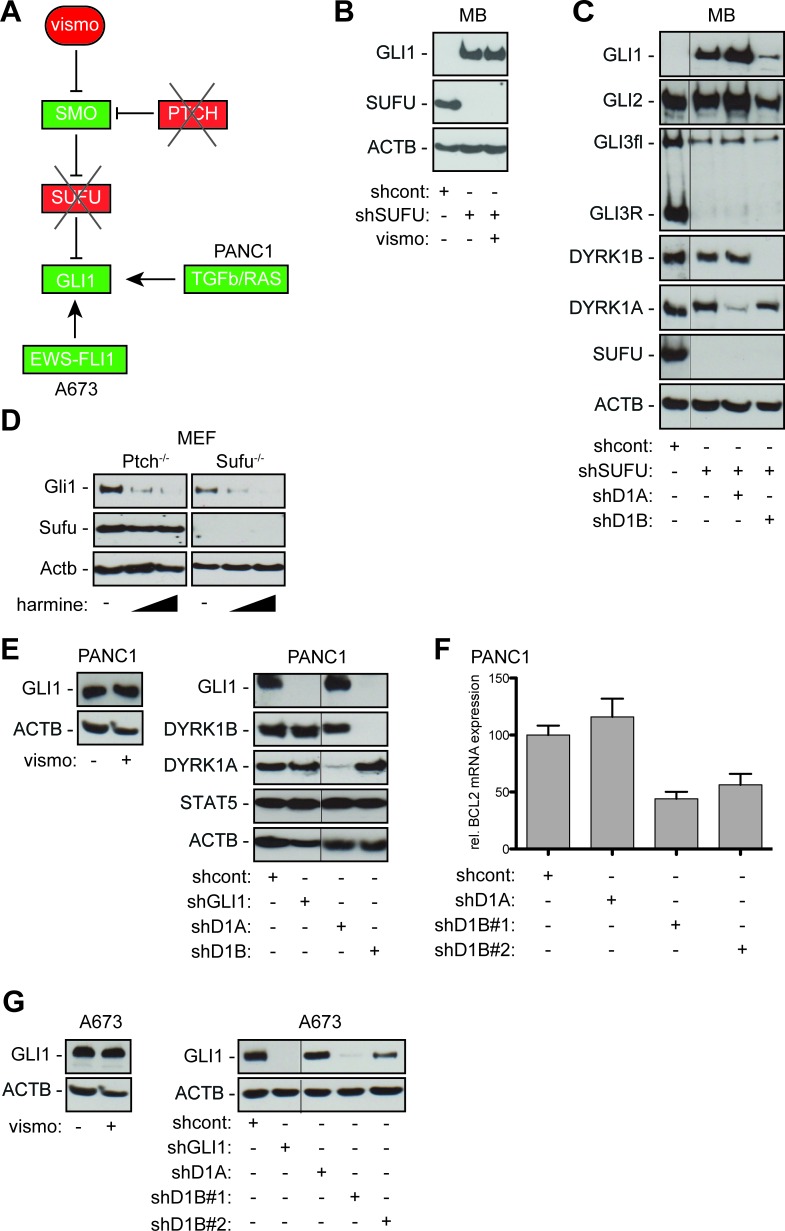
DYRK1B targeting inhibits SMO-dependent and SMO-independent activation of GLI **A**. Illustration of HH/GLI signaling and SMO-targeting in PTCH- or SUFU-deficient cells. In PANC-1 pancreatic cancer cells, TGFb and RAS control GLI1 expression independent of SMO. In Ewing sarcoma cells (A673) the EWS-FLI1 oncoprotein directly activates GLI1 expression. **B**. shRNA-mediated depletion of SUFU renders DAOY cells resistant to SMO inhibition by vismodegib (vismo). Western blot analysis showing that stable expression of shRNA against SUFU (shSUFU) results in activation of GLI1 expression. Note that GLI1 expression in SUFU depleted cells is resistant to SMO inhibition by vismodegib. shcont: scrambled control shRNA. **C**. Western blot showing GLI1, GLI2 and GLI3 expression in wild-type and SUFU-deficient DAOY cells in response to DYRK1A/B knock-down. Note that RNAi against DYRK1B (shD1B) but not against DYRK1A (shD1A) strongly reduces GLI1 and moderately reduces GLI2 expression in SUFU-depleted (shSUFU) DAOY cells. GLI3 expression and processing are unaffected by DYRK1A and DYRK1B targeting. **D**. Harmine treatment (10 μM and 20 μM) inhibits Gli1 protein expression in both Ptch-deficient and Sufu-deficient mouse embryonic fibroblasts (MEF). **E**. Human pancreatic adenocarcinoma cells PANC-1 express detectable levels of GLI1 protein in response to TGFb/SMAD and RAS signaling [23]. GLI1 expression in PANC-1 cells is independent of SMO activity since vismodegib (vismo) treatment does not affect GLI1 protein levels (left panel). RNAi against GLI1 (shGLI1) and against DYRK1B (shD1B) but not against DYRK1A (shD1A) efficiently represses GLI1 protein expression. DYRK1B targeting does not affect non-HH/GLI effectors such as STAT5 (or STAT3 and CTNNB, data not shown). **F**. qPCR analysis of PANC-1 cells showing that inhibition of DYRK1B (shD1B#1, shD1B#2) but not of DYRK1A (shD1A) reduces expression of the GLI target BCL2. **G**. GLI1 expression in the Ewing sarcoma cell line A673 harboring the EWS-FLI1 oncogene is resistant to SMO inhibition by vismodegib (vismo) treatment (left panel). While shRNA against DYRK1A (shD1A) does not affect GLI1 expression in A673 cells, knock down of DYRK1B with two distinct shRNAs (shD1B#1, shD1B#2) decreases GLI1 expression (right panel). shGLI1 knockdown demonstrates specificity of the anti-GLI1 antibody used for detection of GLI1 in PANC-1 and A673 cells. ACTB/Actb: human/mouse beta actin loading control. Fine black lines indicate cropping of intermediate lanes from the same Western blots.

Overcoming *de novo* and acquired resistance to SMO inhibitors is a major challenge in the treatment of HH-associated cancers, underlining the high medical need for HH inhibitors acting independent of SMO. To map DYRK1B within the HH/GLI signaling cascade, we performed epistasis experiments by using PTCH and SUFU-deficient cells displaying SMO-dependent and SMO-independent pathway activation, respectively. In addition, we analyzed the role of DYRK1B in the regulation of GLI1 expression in pancreatic cancer and Ewing sarcoma cells, where TGFβ/RAS and the EWS-FLI1 oncogene, respectively, control GLI1 expression independent of canonical SMO activity (Figure 3A) [23, 24].

As a first approach we triggered SMO-independent GLI activation in human medulloblastoma cells (DAOY) by RNAi mediated knockdown of SUFU, a critical negative GLI regulator acting downstream of SMO (Figure 3A) [41]. As shown in Figure 3B, SUFU-knockdown in DAOY cells led to activation of GLI1 expression that was resistant to vismodegib treatment. In contrast to SMO inhibition, RNAi-mediated depletion of DYRK1B but not of DYRK1A largely prevented GLI1 expression and moderately reduced GLI2 expression while leaving levels and processing of GLI3 unchanged (Figure 3C).

We confirmed the role of Dyrk1b in the regulation of Gli1 using mouse embryonic fibroblasts (MEF) deficient in either *Ptch* or *Sufu*. In line with Dyrk1b acting downstream of Smo, Dyrk1b inhibition by harmine treatment reduced Gli1 expression in *Ptch^−/−^* and *Sufu^−/−^* MEF (Figure 3D), while vismodegib abolished Gli1 expression only in *Ptch^−/−^* but not in *Sufu^−/−^* cells (data not shown).

In light of these findings we hypothesized that targeting DYRK1B may represent a novel strategy to inhibit oncogenic GLI1 activity in cancer cells with non-canonical, SMO-independent GLI1 activation. As proof of concept we tested this hypothesis in pancreatic cancer and Ewing sarcoma cell models. Pancreatic cancer cells have been shown to express GLI1 in response to TGFβ and RAS signaling independent of SMO activation [23, 42]. To test whether DYRK1B contributes to the control of GLI1 expression in pancreatic cancer cells, we analyzed PANC-1 cells for GLI1 expression in response to DYRK1 inhibition. PANC-1 cells display high levels of DYRK1B and express detectable levels of GLI1 protein. GLI1 protein levels remained unaffected upon vismodegib treatment, indicating SMO-independent GLI1 regulation and *de novo* resistance to SMO targeting (Figure 3E, left panel). Of note, RNAi targeting of DYRK1B but not of DYRK1A resulted in loss of GLI1 protein expression and in reduced mRNA levels of the GLI1 target BCL2 [43] (Figure 3E right panel and 3F).

Ewing sarcoma cells harboring the EWS-FLI1 oncogenic fusion gene have been shown to express GLI1 [44] in response to direct binding of the EWS-FLI1 oncoprotein to the GLI1 promoter. Consequently, EWS cells (here A673 cells) display SMO-independent GLI1 expression that is resistant to vismodegib treatment [24] (Figure 3G, left panel). In agreement with the previous data in SUFU-deficient and pancreatic cancer cells, inhibition of DYRK1B but not of DYRK1A reduced GLI1 protein levels (Figure 3G, right panel). Together, these data identify DYRK1B as possible therapeutic target to overcome SMO-inhibitor resistance in GLI1-dependent cancer cells.

### A novel DYRK1 inhibitor as potent antagonist of HH/GLI signaling

Having identified DYRK1B as novel drug target for the inhibition of oncogenic HH/GLI signaling, we set out to screen for small molecule inhibitors with potent activity against DYRK1B and pharmacological properties suitable for *in vivo* administration and therapy.

**Figure 4:**
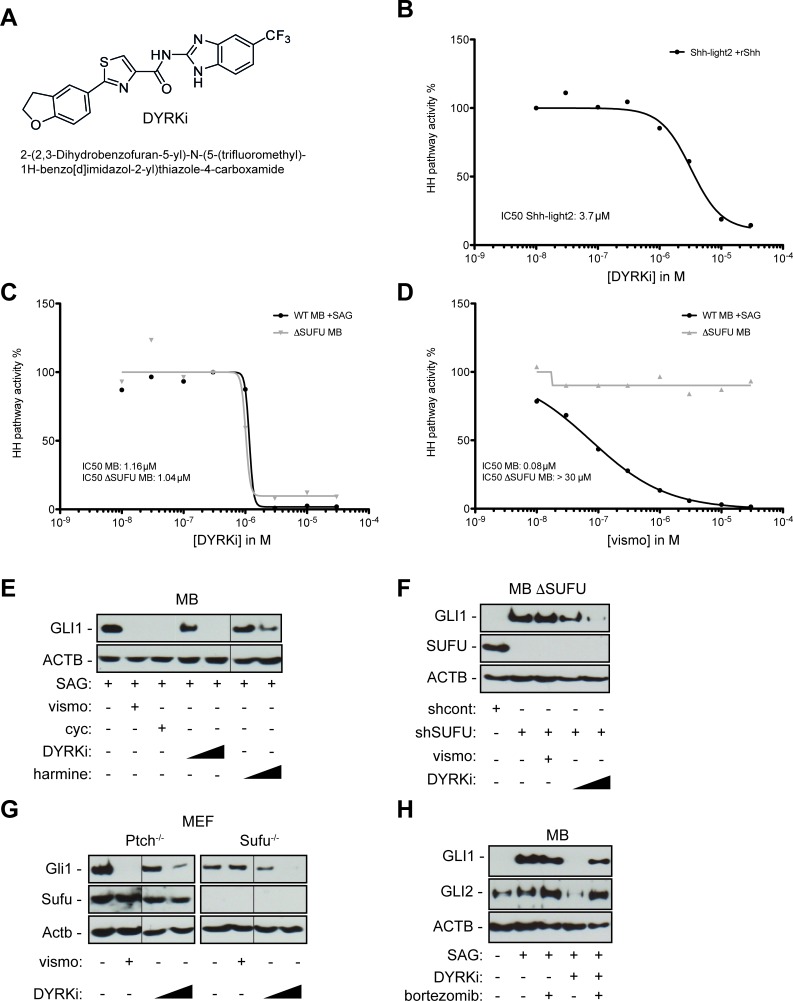
A novel DYRK1 inhibitor efficiently repressing SMO-dependent and SMO-independent GLI1 expression **A**. Chemical structure of DYRKi, a novel DYRK1 inhibitor. **B**. Concentration-dependent inhibition of Hh/Gli signaling in murine Gli luciferase reporter cells by DYRKi resulting in an IC_50_ of 3.7 μM. **C**. DYRKi efficiently blocks HH pathway activity in both SAG-stimulated wild-type human medulloblastoma cells (DAOY, WT MB +SAG) and SMO-inhibitor resistant, SUFU depleted medulloblastoma cells (ΔSUFU MB). **D**. shRNA mediated depletion of SUFU expression renders SAG-stimulated human medulloblastoma cells resistant to SMO inhibition by vismodegib (vismo). Data in C and D were calculated as a function of GLI1 mRNA expression in the respective samples. GLI1 mRNA expression was determined by qPCR. GLI1 mRNA levels of SAG-treated/solvent controls (wild-type DAOY) or solvent-only treated SUFU depleted DAOY cells were set to 100 percent. **E**. GLI1 protein expression in SAG-stimulated DAOY medulloblastoma cells treated with SMO-antagonists vismodegib (vismo, 0.5 μM), cyclopamine (cyc, 5 μM) or with DYRK1 inhibitors DYRKi (1 μM and 5 μM) or harmine (10 μM and 20 μM). **F**. Inhibition of GLI1 protein expression in SUFU depleted DAOY medulloblastoma (shSUFU) cells by DYRKi treatment (1 μM and 5 μM). Note that vismodegib (vismo, 0.5 μM) fails to reduce GLI1 expression. shcont: scrambled control shRNA. **G**. DYRKi treatment (1 μM and 5 μM) represses Gli1 protein expression in both Ptch-deficient and Sufu-deficient mouse embryonic fibroblasts isolated from Ptch- and Sufu knockout mice, respectively. Vismodegib (vismo) inhibits Gli1 expression in Ptch-deficient cells only. **H**. Treatment with the proteasome inhibitor bortezomib largely reverses the suppressive effect of DYRKi on GLI1 and GLI2 protein expression, supporting a model of post-translational regulation of GLI1 and GLI2 stability by DYRK1B. Fine black lines indicate cropping of intermediate lanes from the same Western blots. ACTB/Actb: human/mouse beta actin loading control.

To identify inhibitors of HH signaling we screened in house kinase inhibitors for activity in Shh-light2 reporter gene assay. This led to the identification of a novel DYRK1 inhibitor referred to as DYRKi (Figure 4A) that inhibited reporter gene activity with an IC_50_ of 3.7 μM (Figure 4B) without affecting viability and inhibited the target kinase DYRK1B with an IC_50_ of 90 nM in extracellular *in vitro* ATP competition assays using recombinant protein (data not shown). The compound was shown to be nontoxic, and demonstrated favorable selectivity against other kinases in the DiscoveRX KINOMEScan (selectivity factor S (35)=0.078) (Figure S3 A-D). DYRKi demonstrated *in vivo* a dose proportional exposure of up to 100 mg/kg. Administration of 100 mg/kg resulted in a plasma concentration of ∼10 μM over 8 h, and a terminal t_1/2_ of ∼3 h, demonstrating suitable pharmacokinetics for once daily dosage (Figure S3 A, B).

Importantly and in line with our DYRK1B RNAi data, DYRKi treatment of SAG-treated SUFU-positive (WT MB +SAG) and untreated SUFU-depleted (ΔSufu MB) human medulloblastoma cells prevented GLI1 mRNA expression at comparable IC_50_ concentrations of 1.16 μM and 1.04 μM, respectively (Figure 4C). By contrast, vismodegib treatment prevented GLI1 expression only in SUFU-positive cells but not in SUFU-depleted SMO-inhibitor resistant cells (Figure 4D). DYRKi-treated cells were viable and fully responsive to non-HH stimuli such as EGF, indicating that DYRKi-mediated HH-pathway inhibition is not due to unspecific cytotoxic effects of the compound (Figure S4). Furthermore, DYRKi treatment did not significantly change DYRK1B protein levels in DAOY, PANC-1 or A673 cells (Figure S5). Analysis of GLI1 protein expression in SUFU-positive and SUFU-deficient human medulloblastoma cells (Figure 4E, 4F) and Sufu knockout mouse embryonic fibroblasts (Figure 4G) further corroborated the potent activity of DYRKi to inhibit GLI activity in SMO-inhibitor sensitive and resistant cells. Notable, addition of the clinically approved proteasome inhibitor bortezomib largely reversed the negative effect of DYRK1 inhibition on GLI1 and GLI2 by DYRKi (Figure 4H), suggesting that DYRK1B plays a critical role in preventing the GLI activator forms GLI1 and GLI2 from proteasome-mediated degradation, consistent with the documented role of DYRK1B in protein stabilization [34]. Since we were unable to show direct phosphorylation of GLI1 by DYRK1B (data not shown), the detailed mechanisms of GLI1 (and GLI2) protein stabilization by DYRK1B remain unclear.

### DYRK1B targeting impairs oncogenic growth of GLI-dependent pancreatic cancer cells

**Figure 5:**
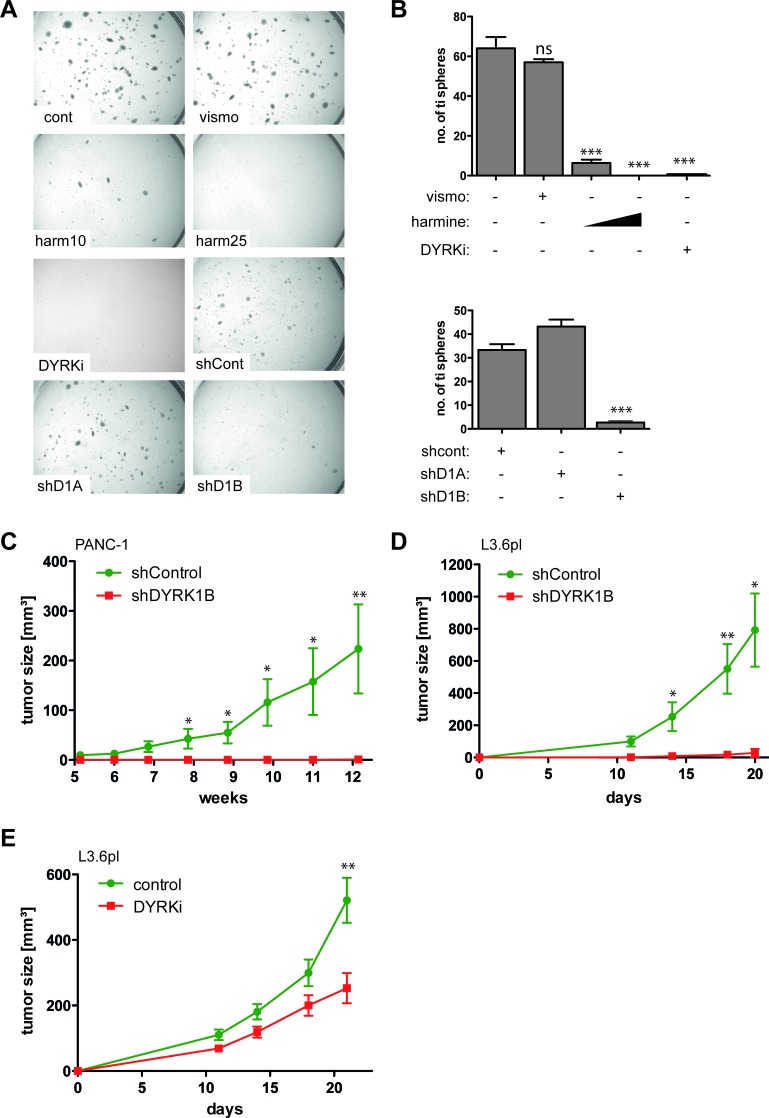
DYRK1B targeting inhibits the malignant properties of GLI1-dependent human pancreatic cancer cells **A**.-**B**. Growth of GLI1 expressing PANC-1 spheres in 3-D cultures. Sphere formation is resistant to SMO inhibition by vismodegib (vismo) and shRNA against DYRK1A. By contrast, harmine (10 μM and 25 μM), DYRKi (5 μM) and shRNA against DYRK1B (shD1B) efficiently prevent the formation of tumor-initiating spheres. Sphere formation shown in A) was quantitatively analyzed and the number of tumor-initiating (ti) spheres plotted in B). For all experiments, identical numbers of live cells were seeded into 3D matrix cultures. We noted that inhibition of DYRK1B does not simply induce cell death but prevents the formation of large spheres formed by highly clonogenic, putative tumor-initiating cells [16]. **C**.-**D**. Xenograft analysis of *in vivo* tumor growth of PANC-1 (n=7) in **C**. and highly metastatic GLI1-dependent L3.6pl pancreatic cancer cell lines (n=6) in **D**. shControl: cells lentivirally transduced with scramble control shRNA, shDYRK1B: cancer cells stably expressing shRNA against DYRK1B. **E**. Oral administration of DYRKi (100 mg/kg/d) significantly reduces *in vivo* tumor growth of GLI1-dependent pancreatic cancer cells (L3.6pl) (n=20). Control mice (n=8) received solvent only (control). * p ≤ 0.05, ** p ≤ 0.01, *** p ≤ 0.001;

In previous work, others and we have provided evidence for a critical tumorigenic role of GLI1 in pancreatic cancer cells, including reduced formation of tumor-initiating spheroids *in vitro* and impaired *in vivo* tumor growth [45-47]. Having shown that GLI1 expression in PANC-1 cells depends on DYRK1B, we therefore addressed whether inhibition of DYRK1B is able to phenocopy the anti-tumorigenic effect of GLI1 inhibition in pancreatic cancer cells. We first monitored the effect of DYRK1B inhibition on tumor-initiating spheroid formation of GLI1-dependent yet SMO-inhibitor resistant PANC-1 cells in 3D cultures [46]. As shown in Figure 5A and 5B, treatment with the SMO inhibitor vismodegib did not affect clonogenic growth, while harmine and DYRKi treatment significantly reduced large spheroid formation. Pre-treatment of pancreatic cancer cells with DYRKi or vismodegib prior to seeding into 3D cultures for clonogenic growth assays yielded similar results. Again, DYRKi reduced the formation of tumor-initiating spheres while vismodegib did not (Figure S6). In agreement with the critical role of DYRK1B in GLI1 expression, RNA-interference against DYRK1B but not against DYRK1A inhibited clonogenic growth of GLI1-dependent pancreatic cancer cells. Note that neither chemical nor genetic inhibition of DYRK1B significantly reduced viability of cells in planar cultures and equal numbers of viable cells were used in all assays.

To test the *in vivo* relevance of these data, we performed xenograft assays with two distinct GLI1-dependent pancreatic cancer cell lines (PANC-1 and L3.6pl) [46]. In line with *in vitro* data, stable knockdown of DYRK1B effectively abolished the engraftment and *in vivo* tumor growth of PANC-1 and L3.6pl pancreatic cancer cells (Figure 5C, 5D). In addition, oral administration of DYRKi significantly reduced *in vivo* tumor growth of pancreatic cancer cells (Figure 5E).

Taken together, these results demonstrate that inhibition of DYRK1B efficiently represses GLI1 expression and reduces the malignant properties of GLI1 dependent cancer cells including pancreatic cancer cells with resistance to SMO inhibitors.

## DISCUSSION

The widespread aberrant activation of HH/GLI in human cancers and its causal role in tumor initiation and growth explain the attractiveness and rationale of targeting HH/GLI signaling in cancer. Most efforts to identify selective HH pathway inhibitors have so far concentrated on targeting the essential pathway effector SMO. In 2012, the FDA approved the first-in class oral SMO inhibitor vismodegib for the treatment of advanced and metastatic basal cell carcinoma with striking therapeutic efficacy, though severe side effects and the rapid development of acquired SMO inhibitor resistance pose significant limitations to the clinical application of drugs targeting SMO [10, 11, 48]. Furthermore, clinical trials on colorectal, ovarian and pancreatic cancer failed to demonstrate therapeutic efficacy of SMO inhibition [13], which may at least in part be due to SMO-independent activation of oncogenic GLI activity by for instance TGFβ, RAS, PI3K/AKT/S6K or genetic deletion of the GLI repressor SUFU (reviewed in [7, 49]). Together, these potential shortfalls of SMO inhibitors call for the identification of targets regulating oncogenic GLI downstream of SMO.

In the present study, we identified DYRK1B as cell-autonomous positive regulator of GLI activity. We have shown that genetic and chemical perturbation of DYRK1B represses the expression of the GLI1 oncogene in a variety of settings, including human brain and pancreatic cancer and murine basal cell carcinoma cells. Importantly, DYRK1B targeting abolished GLI1 expression in SMO-inhibitor sensitive and SMO-inhibitor resistant cells including SUFU deficient medulloblastoma, GLI1-dependent pancreatic cancer [16, 23] and Ewing sarcoma cells expressing GLI1 in response to the EWS-FLI1 oncoprotein [24, 44]. This places DYRK1B function downstream of SMO/SUFU to promote GLI1 oncogene expression.

Mechanistically, we propose that D1B enhances the stability of GLI activator forms [50], as inhibition of the proteasome machinery neutralizes the negative regulatory effect of DYRK1B targeting on GLI1/2 expression. Whether stabilization of GLI1 and GLI2 involves direct phosphorylation by DYRK1B or depends on alternative indirect mechanisms is unclear at present and requires future in-depth analysis of post-translational GLI modifications.

In light of the disappointing outcomes of several clinical trials with SMO inhibitors, targeting oncogenic GLI transcription factors downstream of SMO has emerged as promising alternative therapeutic strategy. Other non-SMO Hedgehog pathway inhibitors including kinase inhibitors or epigenetic modifiers [19, 31, 51], inhibitors of GLI DNA binding and/or post-transcriptional GLI activation [28, 30, 52-54], or GLI antagonists with yet unidentified targets have recently been reported [29], supporting the promising therapeutic potential of targeting oncogenic GLI transcription factors in settings where SMO-inhibition is inefficient. However, except for arsenic trioxide and pyrvinium [51, 52, 54], lack of suitable pharmacological properties, limited potency or toxicity is a concern for many of the GLI antagonists identified. The identification of DYRKi as a novel DYRK1 inhibitor with suitable pharmacokinetics and anti-GLI1 activity provides proof-of-concept for oncogenic HH/GLI inhibition by DYRK1B targeting in malignancies with *de novo* or acquired SMO inhibitor resistance.

Previous studies have already highlighted the complex regulatory role of DYRK family members in HH/GLI signaling. For instance, DYRK2 can directly phosphorylate GLI2 thereby promoting its degradation, while overexpression of DYRK1A can enhance GLI transcriptional activity. DYRK1B, the closest homologue of DYRK1A, can act downstream of RAS to prevent autocrine and promote paracrine HH signaling in RAS mutant cancer cells [35]. The same study has also shown a repressive effect of oncogenic RAS and its downstream effector DYRK1B on GLI expression, contrasting our findings and those of other independent studies that support a GLI-activating role of DYRK1B and RAS, respectively [23, 42, 55]. Whether this discrepancy is due to different experimental conditions (e.g. serum, confluency, cell line passages, transient versus stable long-term knockdown approaches) is unclear.

Our findings that DYRK1B rather than DYRK1A acts as critical positive regulator of HH/GLI add another example of HH/GLI regulation by DYRK kinases with possible therapeutic relevance and also highlight the highly complex and context-dependent control of HH/GLI activity [7].

Using SMO-inhibitor resistant pancreatic cancer cells we also confirm a previous report showing that DYRK1B targeting in pancreatic cancer cells has pronounced therapeutic efficacy *in vitro* and *in vivo*, though the molecular mechanisms including the link to HH/GLI signaling remained unidentified in this study [56]. In the present study we show that interfering with DYRK1B dramatically impairs SMO-independent GLI1 expression in RAS mutant pancreatic cancer cells and phenocopies the anti-tumorigenic effect of GLI1 targeting [16]. Since GLI activator function is critical for tumor-initiating cells [16, 25, 25, 57] and also involved in the development of Ras-driven murine pancreatic cancer within the epithelial tumor compartment [23, 47, 55], we speculate that chemical DYRK1B inhibition may be able to repress the growth of pancreatic cancer due to negative regulation of SMO-independent GLI1 in tumor and tumor-initiating cells, consistent with the abrogation of tumor engraftment in response to genetic DYRK1B inhibition. Intriguingly, up to 10 percent of pancreatic cancers harbor genomic amplifications of DYRK1B and display DYRK1B overexpression in the epithelial compartment of the tumors [33]. Targeting DYRK1B may therefore overcome the inefficient therapeutic response of pancreatic cancer patients to SMO-inhibitors that target canonical paracrine signaling in the tumor environment rather than non-canonical GLI activity in the epithelial tumor compartment [58-61]. It will be important to address in future studies whether DYRK1B overexpression and amplification correlate with increased epithelial GLI1 protein levels and therapeutic response to DYRK1B inhibition in patient samples.

In summary, the identification of DYRK1B as drug target for the inhibition of oncogenic HH/GLI signaling in SMO-inhibitor resistant cancers and the introduction of DYRKi as novel DYRK1B inhibitor with suitable pharmacologic properties provide a basis for future efforts to translate these findings to clinical testing.

## MATERIALS AND METHODS

### Cell culture and inhibitors

DAOY cells (American Type Culture Collection, ATCC, Manassas, VA, USA) were grown as described previously [38]. BSZ2 cells were grown as described in [40] and PANC-1 (ATCC) cells as described in [46].

*Ptch^−/−^* and *Sufu^−/−^* embryonic fibroblasts were grown in DMEM supplemented with 10% fetal bovine serum (FBS), antibiotics and for *Sufu^−/−^*cells also with L-glutamine. During assays and treatments of confluent cells, FBS was reduced to 0.5%. For 3-dimensional (3D) cultures, 5 × 10^3^ cells were seeded in 12-well plates as described previously [62]. 3D spheroid cultures were grown for 4-6 weeks at 37°C in a humidified atmosphere containing 5% CO_2_. Colony formation was documented on a stereomicroscope with Cell^D Image capture system (Olympus, Tokyo, Japan) and quantified using Colony Counter Software (Microtec Nition, Chiba, Japan). Smoothened agonist SAG (Axxora, Farmingdale NY, USA), harmine (Thermo Fisher Scientific, Waltham, MA, USA), vismodegib, cyclopamine (LC Laboratories, Woburn, MA, USA), GANT61 (Merck Chemicals Ltd., Darmstadt, Germany) and DYRKi were dissolved in dimethylsulfoxide (DMSO, Sigma, St. Louis, MO, USA).

### RNA interference and lentiviral transduction

Stable RNAi knockdown experiments were performed by lentiviral shRNA transductions as described in [16]. The following shRNA constructs selected from the Mission TRC shRNA library (Sigma) were used: shRNA DYRK1A (TRCN0000000526), shRNA DYRK1B#1 (TRCN0000002139), shRNA DYRK1B#2 (TRCN0000355722), shRNA DYRK1B#3 (TRCN0000355721), shSUFU (TRCN0000019466) and scrambled control shRNA (SHC002) (Sigma). The shGLI1 construct has been described in [16]. The functionality of shRNAs was validated by Western blot analysis using antibodies listed below.

### RNA isolation, qPCR and Western blot analysis

Total RNA was isolated using TRI-reagent (Molecular Research Center Inc., Cincinnati, OH, USA) followed by LiCl purification. Precipitated and purified RNA was used for cDNA synthesis using Superscript II reverse transcriptase (Life Technologies, Thermo Fisher Scientific) according to the manufacturer's instructions. qPCR was done on a Rotorgene Q (Qiagen, Venlo, Netherlands) using GoTaq qPCR Mastermix reagent (Promega, Fitchburg, WI, USA). HH target genes were identified with primers as described in [16].

For Western blot analysis, proteins were visualized with horseradish peroxidase-conjugated secondary antibodies in combination with enhanced chemiluminescence detection system (GE Health Care, Chalfont St Giles, United Kingdom). The following antibodies were used: anti-GLI1 (V812; Cell Signaling Technology, Danvers, MA, USA), anti-GLI2 (H-300, Santa Cruz Biotechnology, Dallas, TX, USA), anti-GLI3 (R&D Systems, Minneapolis, MN, USA), anti-DYRK1A, anti-DYRK1B (Cell Signaling Technology), anti-SUFU (C-15, Santa Cruz Biotechnology), anti-STAT3 (BD Biosciences, San Jose, CA, USA), anti-STAT5 (3H7, Cell Signaling Technology), anti-Beta Catenin (Cell Signaling Technology), anti-ACTB (Santa Cruz Biotechnology)

### HH/GLI reporter assays

2.5 × 10^4^ Gli Reporter - NIH3T3 cells (AMS Biotechnology Ltd., Abingdon, United Kingdom) were seeded per well into a white 96 well plate. After overnight incubation, compounds were added for 1h prior to HH pathway stimulation with 1 μg/ml murine SHH (R&D Systems). After incubation for 24h the cells were investigated for viability using CellTiter-Fluor^TM^ Kit (Promega) and for reporter gene activity using ONE-Glo™ Luciferase Assay System (Promega).

### Identification and synthesis of the DYRK1 inhibitor DYRKi

We applied the KINOME*scan™* screening platform to quantify interactions between test compounds and more than 450 human kinases and disease relevant mutant variants. The assay was performed at DiscoveRX (San Diego, CA, USA) according to the protocol description available at http://www.discoverx.com/targets/kinase-target-biology. The Image shown in Figure S3 was generated using TREEspot™ Software Tool and reprinted with permission from KINOMEscan®, a division of DiscoveRX Corporation (San Diego, CA, USA).

DYRK1B-Kinase Assay was performed at Reaction Biology Corporation (Reaction Biology Corp., Malvern, PA, USA) according to following protocol. The substrate DYRKtide was prepared in fresh Base Reaction Buffer (20 mM Hepes (pH 7.5), 10 mM MgCl_2_, 1 mM EGTA, 0.02% Brij35, 0.02 mg/ml BSA, 0.1 mM Na_3_VO_4_, 2 mM DTT, 1% DMSO) and required cofactors were added. Recombinant DYRK1B was added to the substrate solution and gently mixed. Compound dilution series in DMSO were added to the reaction, followed 20 min later by addition of a mixture of ATP and ^33^P ATP (specific activity 0.01 μCi/μl final) to a final concentration of 10 μM. Reactions were carried out at 25°C for 120 min, followed by spotting the reactions onto P81 ion exchange filter paper. Unbound phosphate was removed by extensive washing of the filters in 0.75% phosphoric acid. After subtraction of background derived from control reactions containing inactive enzyme, kinase activity data were expressed as the percent remaining kinase activity in test samples compared to vehicle (DMSO) reactions. IC_50_ values and curve fits were obtained using Prism (Graph Pad Software, La Jolla, CA, USA).

Synthesis of DYRKi was done as follows: to a solution of 2-(2,3-dihydro-1-benzofuran-5-yl)-1,3-thiazole-4-carboxylic acid (1.00 g. 4.04 mmol) in 20 ml *N,N*-dimethylformamide, 5-(trifluoromethyl)-1*H*-benzo[*d*]imidazol-2-amine (895 mg, 4.45 mmol), 2-(1*H*-benzotriazole-1-yl)-1,1,3,3-tetramethyluronium hexafluorophosphate (HBTU) (1.53 g, 4.04 mmol), 4-dimethylaminopyridine (49 mg, 0.40 mmol) and *N,N*-diisopropylethylamine (1.76 ml, 10.11 mmol) were added. The reaction mixture was stirred for 72 h at room temperature, poured into ice water and the formed precipitate dried and purified by flash column chromatography (DCM/MeOH 95:5 to 0:100). The crude product was suspended in Et_2_O, filtered and dried. The product was obtained as a white solid (1.07 g, 2.49 mmol, 62 % yield). mp: 232.5; ^1^H NMR (400 MHz, DMSO-*d*_6_) d 12.51 (bs, 1 H), 11.81 (bs, 1 H), 8.50 (s, 1 H), 8.04 (bs, 1 H), 7.85 (dd, *J*=8.33 Hz, *J*=1.91 Hz, 1 H), 7.77 (bs, 1 H), 7.62 (d, *J*=8.34 Hz, 1 H), 7.40 (dd, *J*=8.46 Hz, *J*=1.44 Hz, 1 H), 4.57 (t, *J*=8.75 Hz, 2 H), 3.16-3.30 (m, 2 H); ^13^C NMR (75 MHz, DMSO-*d*_6_) d 168.0, 162.1, 159.9, 148.4, 148.2, 128.6, 127.4, 126.9, 125.8, 125.1, 123.7, 123.3, 122.1, 121.7, 118.0, 118.0, 117.9, 109.2, 71.7, 28.5; analysis (calcd., found for C20H13F3N4O2S): C (55.81, 55.56), H (3.04, 3.17), F (13.24, 13.1), N (13.02, 12.98), S (7.45, 7.18); LC/MS [M+H]^+^: 431.0.

### Xenograft experiments

For *in vivo* tumor growth studies 1 × 10^6^ PANC-1 or 1 × 10^5^ L3.6pl pancreatic cancer cells in 25% Matrigel (BD Biosciences) were injected subcutaneously into the lower flanks of Foxn1^nu^ nude mice (Charles River Laboratories, Wilmington, MA, USA). For *in vivo* treatment, DYRKi was dissolved in sun flower oil and administered at 100mg/kg/d by oral gavage. Tumor volume was measured with a caliper and calculated according to the formula [4/3 × π × (length/2) × (width/2) × (height/2)].

## SUPPLEMENTARY MATERIAL FIGURES


