# Supplementary material for: DYRK1B as therapeutic target in Hedgehog/GLI-dependent cancer cells with Smoothened inhibitor resistance
Source: Oncotarget. 2016 Jan 13;7(6):7134–48. doi: 10.18632/oncotarget.6910 (PMC4872774; doi:10.18632/oncotarget.6910)
Supplement: Supplementary file 1 [file oncotarget-07-7134-s001.pdf]

DYRK1B as therapeutic target in Hedgehog/GLI-dependent cancer cells with Smoothened inhibitor resistance

Supplementary Material

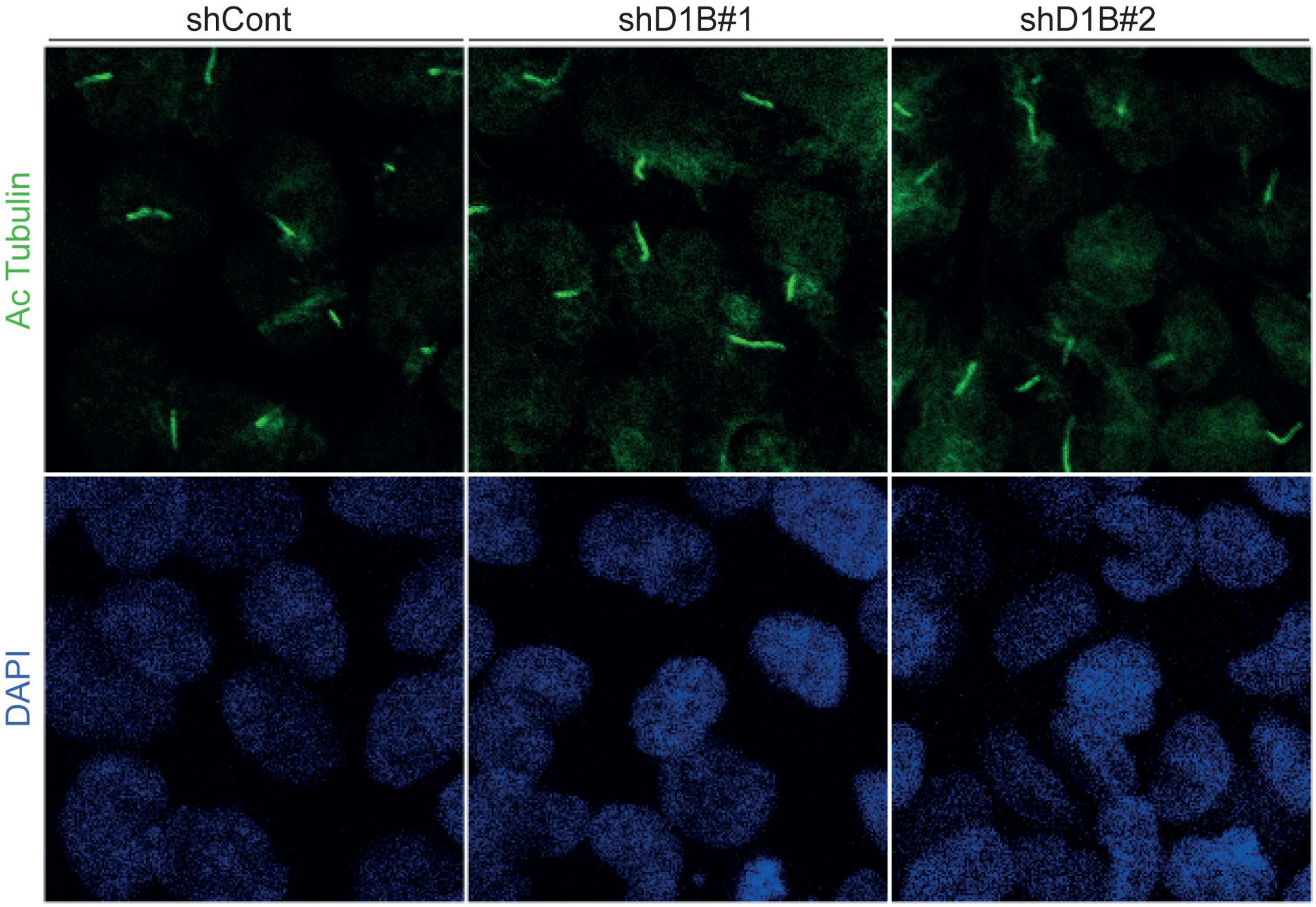

Figure S1: Normal ciliogenesis in DYRK1B depleted human medulloblastoma cells. DAOY cells stably expressing scrambled control (shCont) or two different DYRK1B shRNAs (shD1B#1, shD1B#2) were stained with anti-acetylated tubulin antibodies to visualize the primary cilium. Cells were counterstained with DAPI and analyzed by confocal imaging.

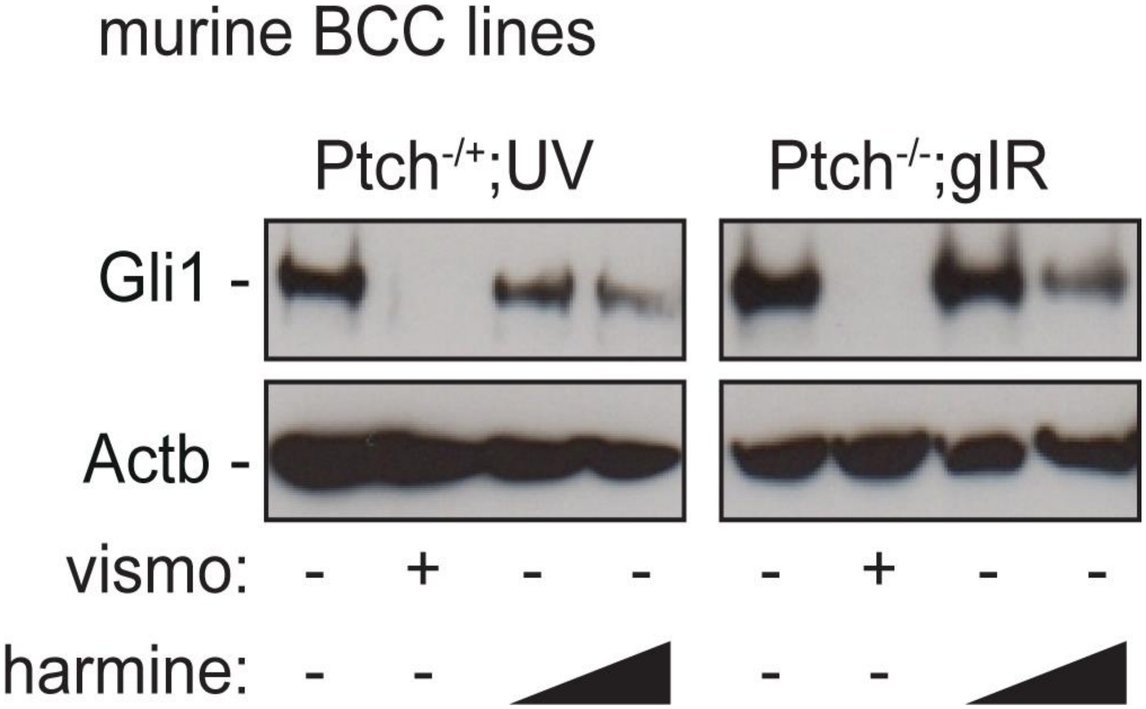

Figure S2: Harmine represses Gli1 expression in murine BCC cell lines. BCC cell lines derived from either UV-irradiated or gamma-irradiated Ptch mutant mice (So, Langston et al. 2006) were treated with vismodegib (0.5 μM) or harmine (5 μM and 10 μM) and Gli1 expression analyzed by Western blotting. vismo: vismodegib; Actb: beta actin loading control.

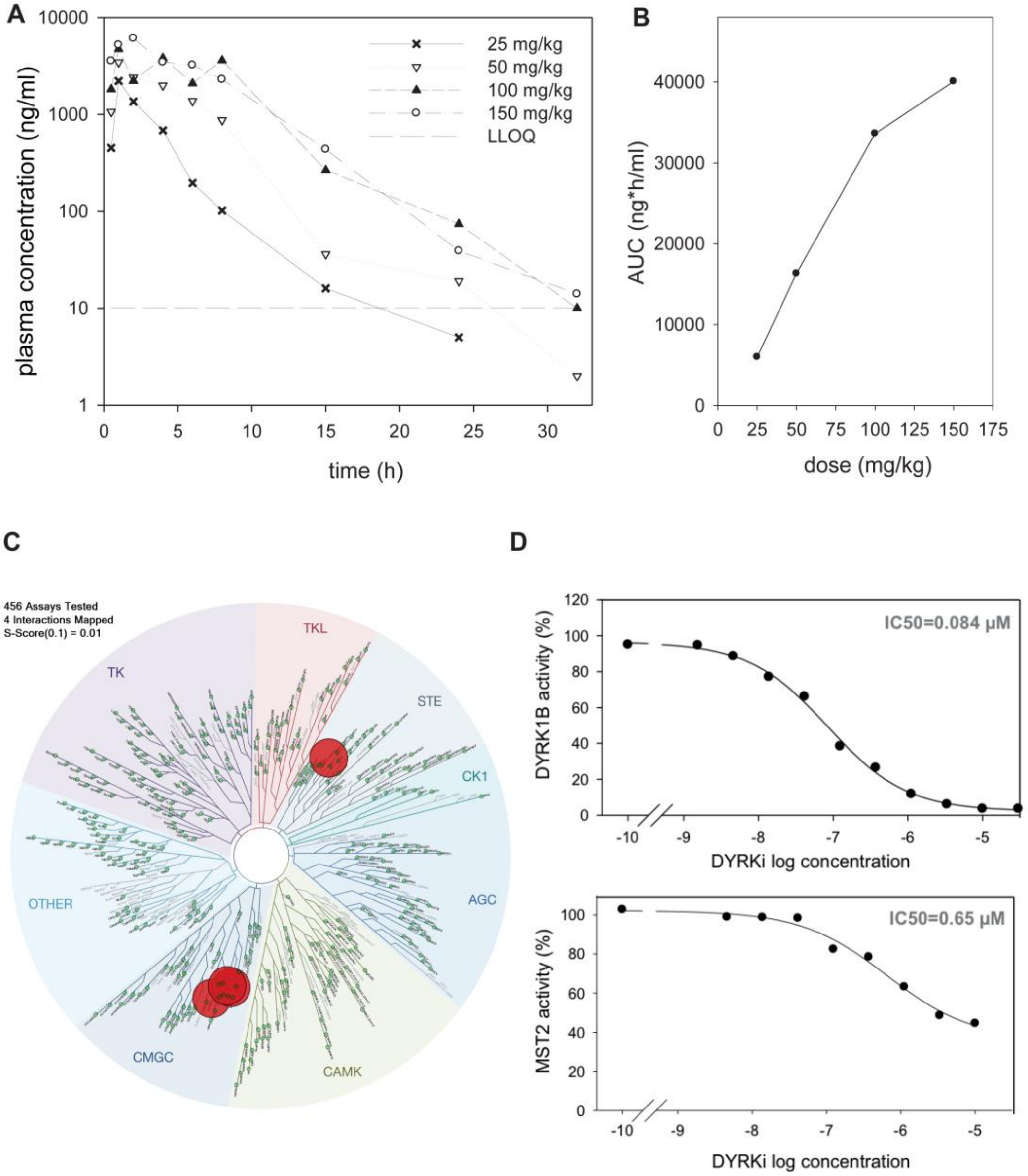

Figure S3: Pharmacologic properties of DYRKi. A) Pharmacokinetic profile of DYRKi after a single oral administration at a dose of 25 mg/kg, 50 mg/kg, 100 mg/kg and 150 mg/kg was determined in female SWISS mice. B) Medium AUCinf versus dose level diagram demonstrates not proportional increase from 100 to 150 mg/kg indicating a saturation effect. Kinase specificity of DYRKi was tested using the DiscoveRX KINOME*scan*™ screening platform. This active site-directed competition binding assay measures quantitatively interactions between test compounds and 456 human kinases. DYRKi demonstrated very high selectivity and yielded only a limited number of hits with more than 99% inhibition. In addition to DYRK1B-related CMGC kinases (i.e. DYRK1B, DYRK1A, CLK1/2, shown in red circles) only MST2 (red circle in STE family) was hit (C). To assess the functional significance of this interaction, DYRKi activity on DYRK1B and MST2 was tested. As shown in D) DYRKi effectively inhibited DYRK1B activity with an IC50 of ~84 nM, while MST2 was only partially inhibited at an IC50 of 650 nM.

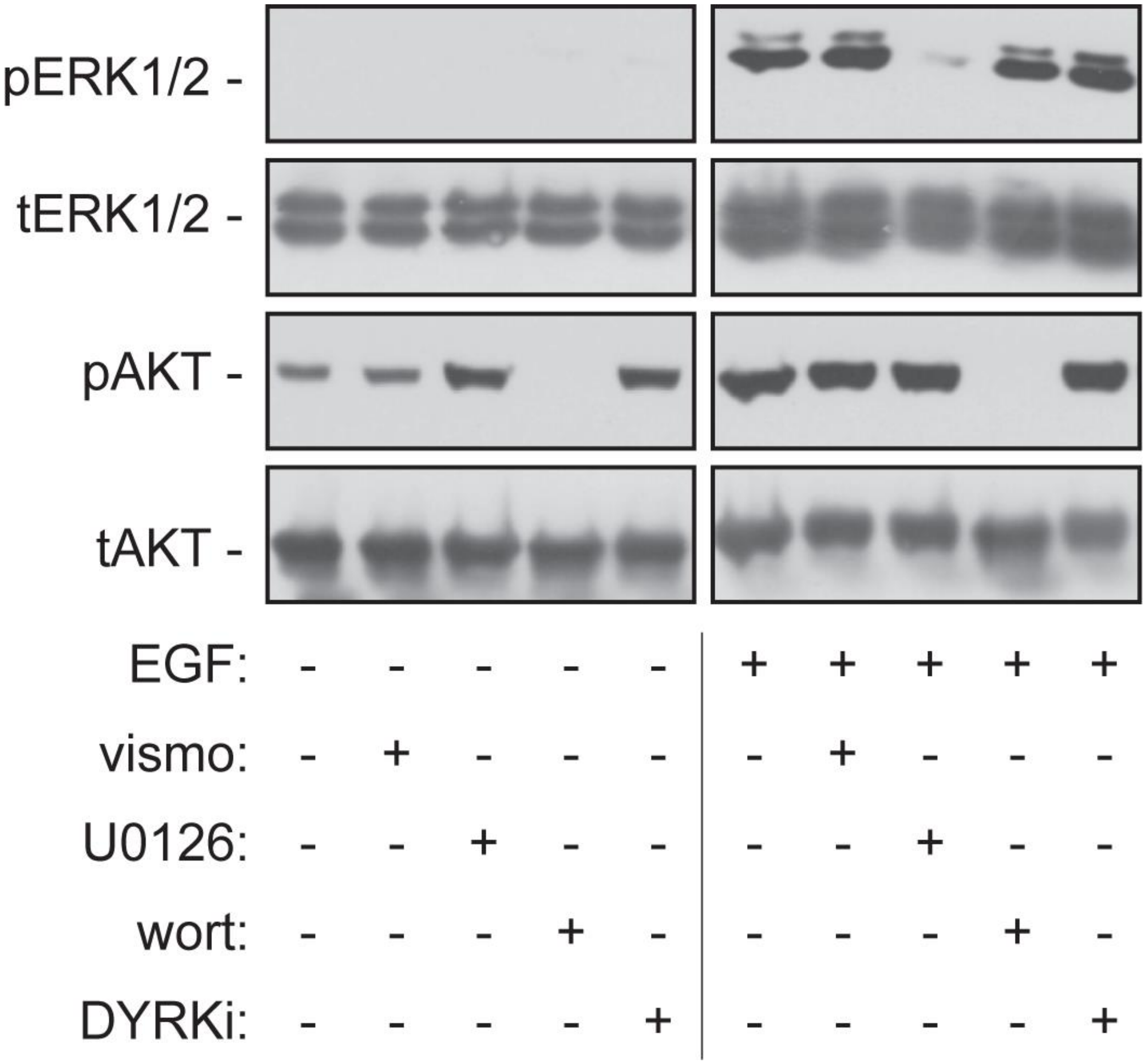

Figure S4: DYRK1B targeting by DYRKi treatment does not result in general defects in cell signaling. Human medulloblastoma cells (DAOY) were stimulated with EGF or left unstimulated and treated with either the SMO inhibitor vismodegib (vismo), MEK1/2 inhibitor U0126, PI3K inhibitor wortmannin (wort) or DYRK1B inhibitor (DYRKi). While DYRKi treatment impairs HH/GLI pathway activation (see main text), DYRKi does not interfere with non-HH signaling such as the EGFR pathway characterized by ERK1/2 (pERK) and AKT (pAKT) activation in response to EGF stimulation. The data also provide evidence that DYRKi leaves cells viable and fully competent to respond to non-HH stimuli.

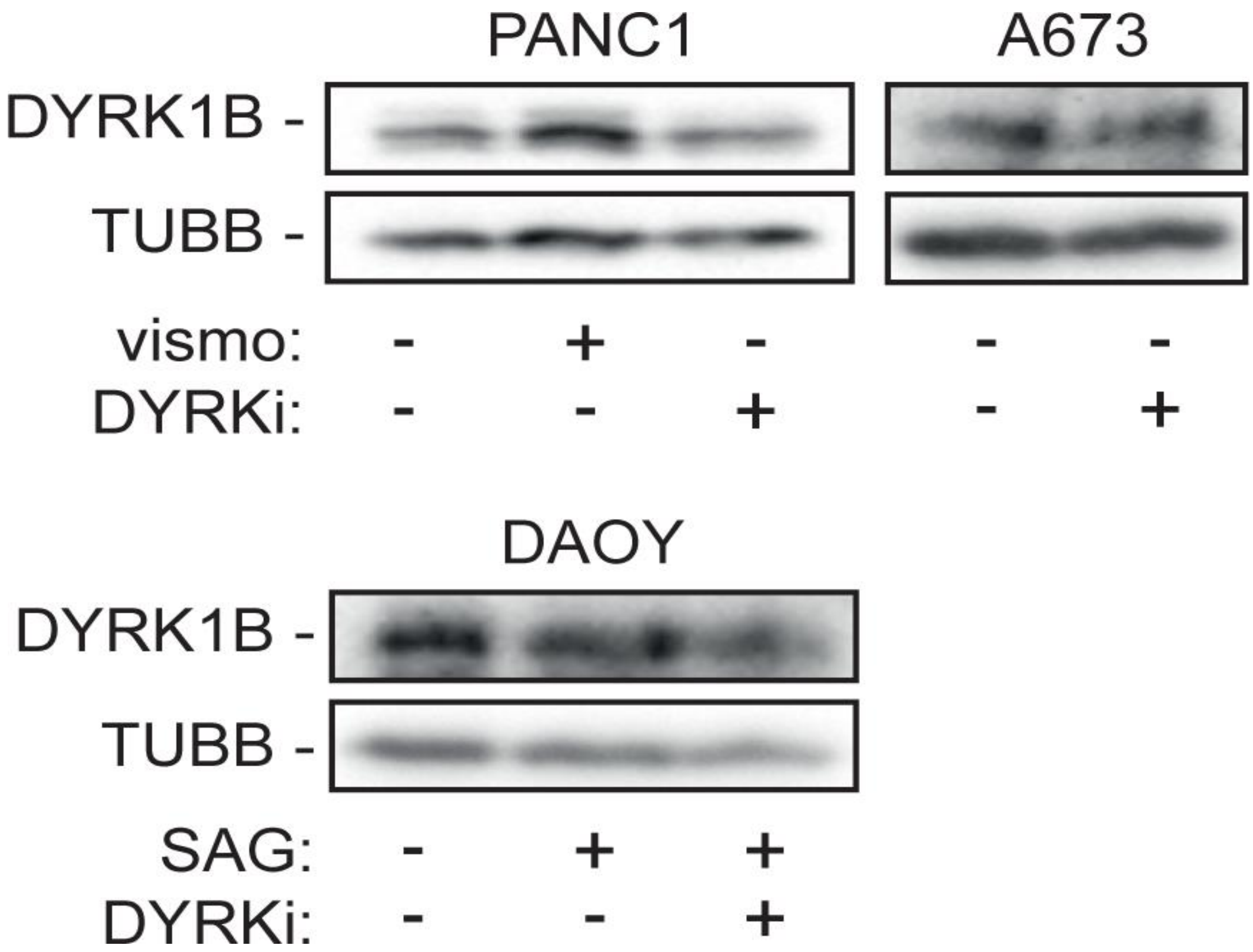

Figure S5: DYRKi treatment does not change total DYRK1B levels. PANC-1, A673 and DAOY cells were treated for 48 h with solvent control (DMSO), Smoothened agonist SAG (100nM)(DAOY), Smoothened antagonist vismodegib (vismo)(PANC-1) or 20µM of DYRKi. DYRK1B protein expression was analyzed by Western blot. Beta tubulin (TUBB) served as loading control.

A

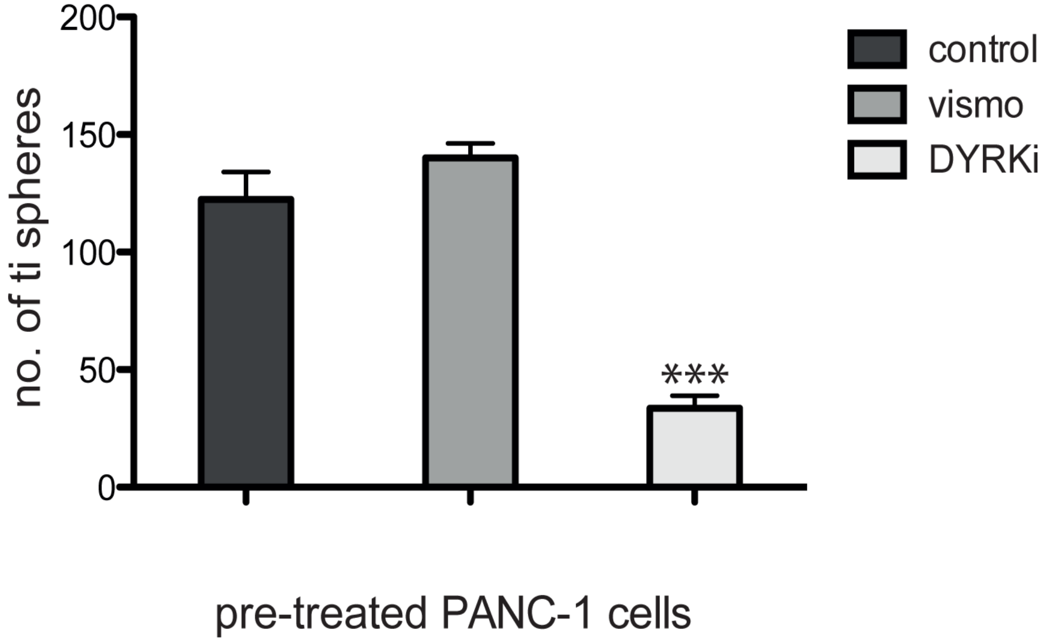

B

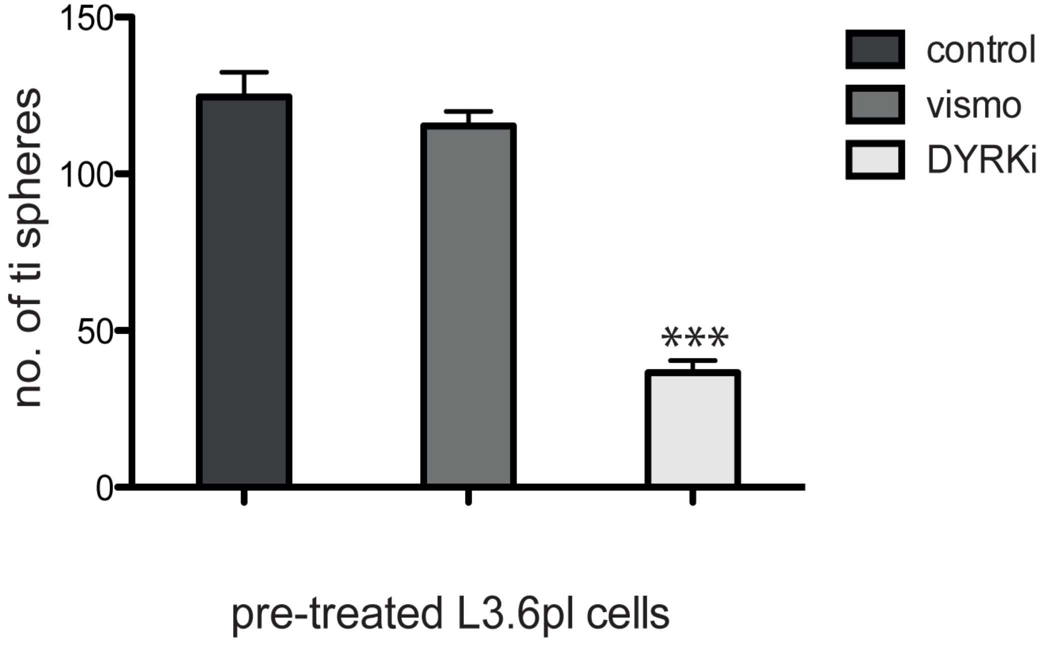

Figure S6: Short-term pre-treatment with DYRKi represses clonogenic growth of GLI1-dependent tumor-initiating pancreatic cancer cells. 1 x 10^4 PANC-1 (A) or 2 x 10^4 L3.6pl cells (B) were pre-treated for 48 h hours with DMSO (control), vismodegib (vismo) (0.5 µM) or DYRKi (20 µM) prior to seeding to 3D cultures for clonogenic growth of tumor-initiating cells. Inhibitors were removed prior to seeding into 3D matrix cultures. ti spheres: tumor-initiating spheres; *** = $p < 0.001$;

So, P. L., A. W. Langston, N. Daniallinia, J. L. Hebert, M. A. Fujimoto, Y. Khaimskiy, M. Aszterbaum and E. H. Epstein, Jr. (2006). "Long-term establishment, characterization and manipulation of cell lines from mouse basal cell carcinoma tumors." Exp Dermatol **15**(9): 742-750.
